# COSMIN guideline for systematic reviews of patient-reported outcome measures version 2.0

**DOI:** 10.1007/s11136-024-03761-6

**Published:** 2024-08-28

**Authors:** Lidwine B. Mokkink, Ellen B.M. Elsman, Caroline B. Terwee

**Affiliations:** 1 Department of Epidemiology and Data Science, Amsterdam UMC, Vrije Universiteit Amsterdam, Location AMC, J1B -225, Meibergdreef 9, Amsterdam, 1105 AZ The Netherlands; 2 Amsterdam Public Health research institute, Methodology, Amsterdam, The Netherlands

**Keywords:** Systematic reviews, Outcome measurement instrument, Patient-reported outcome measures, Measurement properties, COSMIN

## Abstract

**Purpose:**

Systematic reviews of patient-reported outcome measures (PROMs) are important tools to select the most suitable PROM for a study or clinical application. Conducting these reviews is challenging, and the quality of these reviews needs to be improved. We updated the COSMIN guideline for systematic reviews of PROMs, including the COSMIN Risk of Bias checklist, and the COSMIN criteria for good measurement properties.

**Methods:**

Adaptations to the methodology were based on our experience with applying the COSMIN guideline, through discussions among the authors, and results from two related Delphi studies.

**Results:**

The updated guideline places more emphasis on key aspects that are often missing or sub optimally conducted in published systematic reviews of PROMs, such as formulating a well-defined research question and developing a comprehensive search strategy, assessing risk of bias, applying criteria for good measurement properties, summarizing results, and grading the quality of the evidence. We also stress the importance of evaluating the measurement properties of each subscale of a PROM separately and evaluating content validity of all included PROMs.

**Conclusion:**

The quality of systematic reviews of PROMs can be improved by using this updated version of the COSMIN guideline for systematic reviews of PROMs. Improved quality will lead to better PROM selection and increased standardization of PROM use.

**Supplementary Information:**

The online version contains supplementary material available at 10.1007/s11136-024-03761-6.

## Abstract (plain language summary)

Patient-reported outcome measures (PROMs) are questionnaires that measure aspects of health from the patient perspective. To measure a specific health aspect, often dozens of PROMs are available. To choose the best PROM, a systematic review of PROMs can be conducted, in which all information on the quality and feasibility of each available PROM is collected, rated, and compared. Based on such a review a choice for the most suitable PROM for a certain study or clinical application can be made. However, conducting a systematic review of PROMs is very challenging, because nine quality aspects of PROMs need to be taken into account.

In this article, we present an updated step-by-step guideline for conducting systematic reviews of PROMs. Each of these steps is described in detail in an accompanying manual. This updated guideline helps researchers to conduct systematic reviews of PROMs in a systematic and transparent way. It also helps readers of systematic reviews to understand how the review was conducted and to check the conclusions about which PROMs are recommended based on their quality.

**Supplementary Information:**

The online version contains supplementary material available at 10.1007/s11136-024-03761-6.

## Introduction

Systematic reviews of patient-reported outcome measures (PROMs) are important tools to facilitate the selection of PROMs for clinical trials, patient monitoring, or core outcome sets (COS) [1, 2]. One of the biggest challenges of using PROMs in research and clinical practice is the sheer variety of PROMs used, which differ in content and quality and often lack evidence on their measurement properties [3–5]. For example, in a recent systematic review 116 unique PROMs (including 54 disease-specific PROMs) were identified for measuring health-related quality of life in people with diabetes [6]. Only 27% of the disease-specific PROMs were found to have sufficient content validity [7]. Similar results are found in other medical fields (e.g. [8, 9]). This unfavorable situation is a serious threat to the validity of PROM data obtained in research and clinical practice. Suboptimal or even harmful treatment decisions may be made based on invalid PROM data. In addition, the heterogeneity of PROMs used, with mostly incomparable scores, complicates meta-analyses of PROM data and hampers the usefulness of PROMs to inform value-based health care.

Thus, there is an urgent need for better PROM selection and for increased standardization of PROM use. Systematic reviews of PROMs, which critically appraise the measurement properties of all available PROMs that measure a specific construct in a specific population, are essential to achieve this goal. Such reviews provide an overview of the quality of the available PROMs as well as information on their feasibility and interpretability. They identify poor quality PROMs which should not be used, highlight shortcomings in validation research of PROMs, and may identify the need for the development of new PROMs. The number of published systematic reviews of PROMs continues to increase. For example, in the COSMIN database the number of systematic reviews of PROMs increased from less than 10 per year before 2000, to 20–50 per year between 2000 and 2010, up to over 100 per year after 2015 [10].

The mission of the COSMIN initiative is to improve the selection of outcome measurement instruments in research and clinical practice by developing methods and practical tools. In 2018, we published the COSMIN guideline for systematic reviews of PROMs [11]. Conducting systematic reviews of PROMs is challenging, because nine different measurement properties should be evaluated and taken into account when drawing a conclusion on each PROM. For this reason, we also developed two manuals to provide users with many details on how to conduct each step of a systematic review of PROMs [12, 13].

Despite these resources, the quality of conducting and reporting these reviews is still lagging behind. Although we found important improvements in the quality of systematic reviews published in the past decade in a recent study [14], we still found important limitations in the quality of these reviews, especially with respect to the search strategy, risk of bias assessment of the included studies, evaluation of the measurement properties of the included instruments, data synthesis and certainty assessment. To address these limitations, we updated the COSMIN guideline for systematic reviews of PROMs. In this paper, we describe the COSMIN guideline for systematic reviews of PROMs version 2.0. In addition, we updated the COSMIN Risk of Bias checklist, for assessing the quality of studies on measurement properties of PROMs, leading to version 3.0, and the COSMIN criteria for good measurements (version 2.0).

## Methods

In the absence of empirical evidence, and due to a lack of resources, we have updated the COSMIN guideline based mainly on discussions within our author team, rather than a formal structured qualitative study. We updated the guideline based on our experience with conducting systematic reviews [6, 7, 15–17], in assisting other groups in conducting their reviews [e.g. 18–20], as well as discussions in two Delphi studies in which we developed the COSMIN Risk of Bias tool for assessing reliability and measurement error [21], and the PRISMA-COSMIN for Outcome Measurement Instruments (OMIs) reporting guideline [22]. Each step of the guideline was extensively discussed within our group (i.e. among the authors) between March 2022 and October 2023. In addition, the COSMIN guideline for systematic reviews of PROMs is consistent with existing guidelines for other kinds of systematic reviews, such as the Cochrane Handbook for systematic reviews of interventions [23] and for reviews of diagnostic test accuracy [24], and the Grading of Recommendations Assessment, Development and Evaluation (GRADE) principles [25]. A science communication specialist helped us changing the style of the manual to make it more user-friendly.

## Results

Below, we describe how to define the scope of the review, and the eight-steps procedure to conduct the review, and point to supplementary materials to facilitate users of the COSMIN guideline.

### Scope of the review

The scope of a systematic review of PROMs includes four key elements, referring to the construct, the population, the type of instrument, and measurement properties. These key elements should be clearly defined because they are consequential for the research question, the search strategy, inclusion criteria, and generalizability of the results of the review, and will help future users of the review to assess the relevance and comprehensiveness of the review for their purpose. The construct of interest of the review also impacts the evaluation of content validity of the included PROMs. In this guideline we focus on PROMs as the type of instrument. If users also aim to include other types of outcome measurement instruments in their review, such as clinician-reported outcome measures or performance-based tests, some steps should be adapted. For example, when non-PROMs are included, the Risk of Bias tool for reliability and measurement error [21] should be used instead of the boxes Reliability and Measurement error of the COSMIN Risk of Bias checklist for PROMs. If users aim to include non-health related outcomes, such as Patient-Reported Experience Measures (PREMs), or social or behavioral outcomes, such as social support or physical activity, the process will often be same, although we envision that these outcomes are more often based on formative models. For constructs based on formative models, content validity is even more important, while structural validity and internal consistency are not relevant. Last, in some situations it is justified to evaluate only a subset of measurement properties. For example, if the workload of a review seems too high, one could choose to first only evaluate content validity of all PROMs.

### Eight-step procedure

**Fig. 1:**
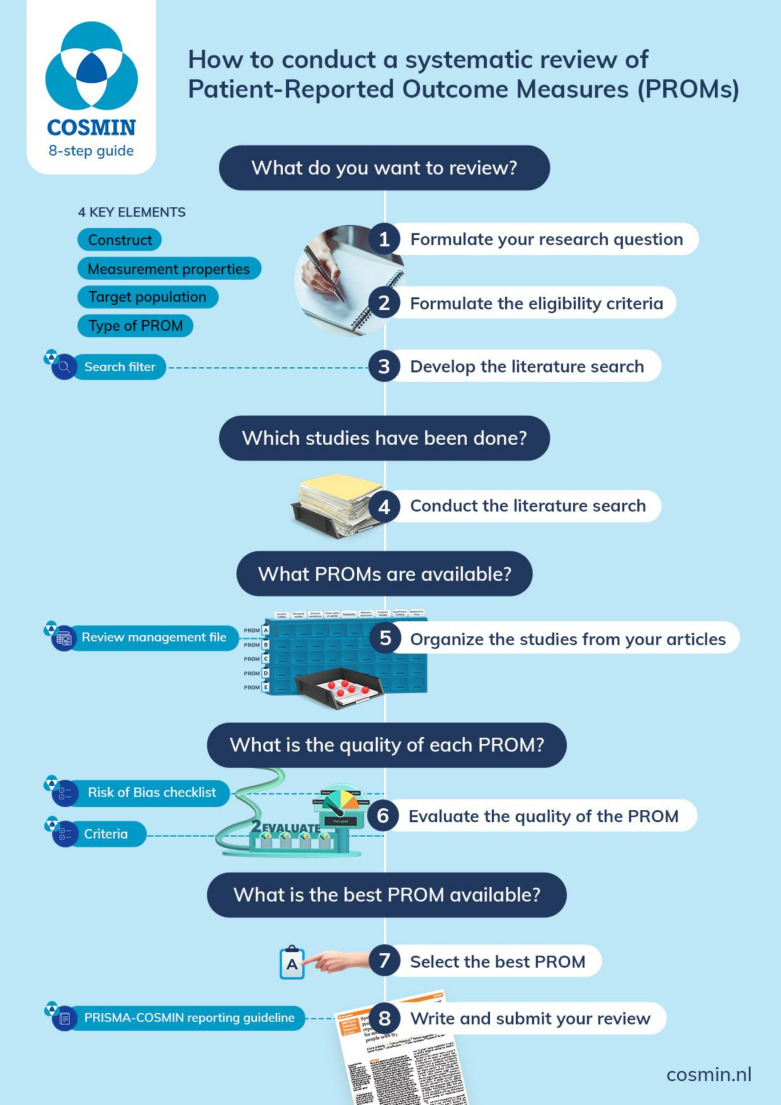
Eight step procedure for conducting a systematic review of a PROM

Once the scope of a review is determined, the process of conducting the review can start. The original ten-step procedure for conducting a systematic review of PROMs was changed to an eight-step procedure (Fig. 1). Each step refers explicitly to what should be done: formulate the research question (step 1), formulate the eligibility criteria (step 2), develop the literature search (step 3), perform the search (step 4), extract the PROM data (step 5), evaluate the nine measurement properties per PROM (step 6), formulate recommendations (step 7), and report the systematic review (step 8). In the animated video these steps are explained.

### Steps 1–3: writing the protocol

In the first three steps, the four key elements related to the scope of the review determine the research question (step 1), followed by the eligibility criteria (step 2) and the search strategy (step 3). The choices made can be described in the protocol of the review, which can be registered in the PROSPERO database. Registering a review or study is common practice of open science, and will strengthen the transparency and credibility of the research.

### Step 4: is the review feasible?

The workload of a systematic review can be high. Therefore, attention should be paid to the feasibility of the review. In step 4 the search is performed, and it becomes clear how many articles will be included. Including 25 articles is doable, while including 50 or more articles may not be feasible, especially when multidimensional PROMs are included and all measurement properties will need to be evaluated for each subscale. A realistic assessment of the amount of work and whether this is feasible must be made to avoid not completing the review or compromising on quality. If it turns out that the amount of work is too high, the research aim could be narrowed, the review could be split (e.g. one review on content validity only, and one on the other measurement properties), or the review team could be expanded. If the number of included articles is very low, the aim could be expanded (e.g. include a broader population), to be able to draw conclusions on the included PROMs.

### Step 5: which PROMs are included?

In step 5 the PROM data is extracted. A PROM is described by its name, version and scope [21]. The scope of a PROM refers to the construct it intends to measure, the origin of the construct, the target population for which it was developed, and the intended context of use (e.g. in clinical trials). Information about the PROM and its scope is required for the evaluation of some measurement properties (e.g. for evaluating content validity and for deciding which comparison instruments could be considered as a reasonable gold standard for criterion validity or relevant for evaluating construct validity). Finally, information on feasibility (such as length, structure, and cost) and interpretability (such as floor and ceiling effects and minimal important change (MIC) values) are extracted, to facilitate the recommendation process.

Often, multiple versions of the same PROM exist, for example, different language versions, versions with a different number of items (e.g. modified versions or short forms), versions that have a different mode of administration, etc. Changes in the response scale or the scoring algorithm also lead to different versions of a PROM. Moreover, if a PROM consists of multiple subscales, each subscale measures a separate aspect of the construct. Each PROM version and each PROM subscale is considered to be a unique PROM, as the measurement properties may be different for each version or subscale. For example, the longer version of a PROM may be comprehensive (which is one aspect of content validity), while the shorter version may lack relevant items. Or, a certain subscale may contain relevant items (another aspect of content validity), whereas another subscale does not. By considering each version and each subscale of a PROM a unique PROM, the ones with the highest quality can be recommended for use in the future.

### Step 6: evaluate the measurement properties of the PROMs

**Fig. 2:**
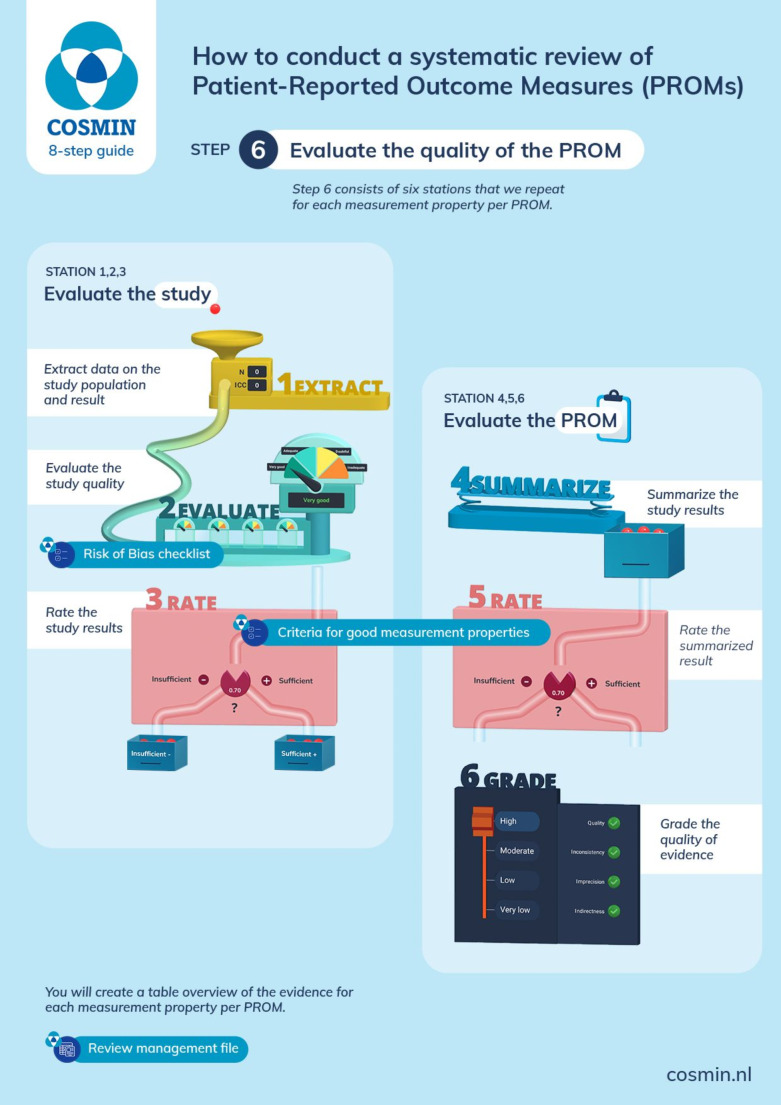
Process for evaluating the measurement properties per PROM

A systematic review of PROMs is challenging, because there are nine different outcomes (i.e. the measurement properties) to evaluate. These measurement properties are evaluated in nine different ways, as each measurement property has its own design requirements and preferred statistical methods. The goal of step 6 of the systematic review is to give an overview of, and summarize all evidence found in the literature per measurement property per PROM. This takes six sub-steps per measurement property per PROM, where first the quality of each *study* is evaluated, and next the quality of each *PROM* is evaluated. Figure 2 shows the process. Below, we describe each sub-step.

We recommend to evaluate the measurement properties one-by-one, i.e. start with evaluating the content validity of all PROMs, then evaluate structural validity and internal consistency, and next, cross-cultural validity\measurement invariance, reliability and measurement error, criterion validity (if applicable), hypotheses testing for construct validity, and responsiveness. We recommend this order for three reasons: (1) some measurement properties are more important than others; (2) some measurement properties are prerequisites for others; and (3) by assessing the measurement properties one-by-one, the assessments will be more consistent across studies and PROMs.

Content validity is considered to be the most important measurement property because the items of the PROM need to be relevant, comprehensive, and comprehensible for the construct of interest and target population [26]. The content validity of all PROMs should be assessed, even when no content validity study is found for a PROM, because information on the PROM development can be used, as well as the reviewer’s own rating of content validity. If there is high-quality evidence that a PROM has insufficient content validity, the other measurement properties need no longer be evaluated because the PROM should not be recommended for use.

### Step 6.1: extract data on study populations, methods, and results

In step 6.1 the characteristics of the study populations of the studies on measurement properties are extracted, such as geographical location, language, and important demographic and disease characteristics, to understand to which underlying population the results refer to. In addition, results on measurement properties are extracted.

### Step 6.2: assess the methodological quality of the studies – is there risk of bias?

An important step in step 6 of a systematic review of PROMs is to assess the risk of bias in a study. Bias can occur in a study on measurement properties when there are important flaws in the design or analysis of the study, which may lead to biased results, such as over- or underestimating the reliability or validity of the PROM. The risk of bias (or methodological quality of a study) should be taken into account when grading the quality of the total body of evidence (certainty assessment) per measurement property per PROM. We updated the COSMIN Risk of Bias checklist. The COSMIN Risk of Bias checklist version 3.0 is provided in Appendix 1. In Appendix 2 we explain and justify the changes compared to the previous version.

### Steps 6.3: rate the study results against criteria for good measurement properties

Each result of an individual study on a measurement property is being compared against the COSMIN criteria for good measurement properties (see Table 1), to decide whether the result is sufficient or not. This facilitates transparent conclusions and recommendations. In Appendix 3 we explain and justify changes we made to the previous set of criteria for good measurement properties [10].

**Table 1:** Criteria for good measurement properties

Measurement property	Rating	Criteria
Content validity	+	Included items are relevant for the construct, target population, and context of use, and response options and recall period are appropriate *AND* No key concepts are missing *AND* PROM items and response options are appropriately worded and PROM instructions, items and response options understood by the population of interest as intended
	?	Not enough information reported
	-	Included items are not relevant for the construct or target population *OR* Key concepts are missing *OR* PROM items and response options are not appropriately worded or not understood by the population of interest as intended
Structural validity	+	**CTT**: EFA/PCA: factor loadings of each item on its factor ≥ 0.30 *AND* Maximum 10% of the items have factor loadings ≥ 0.30 on multiple factors *AND* Explained variance *≥* 50% and structure is in line with the theory about the construct to be measured *OR* results on scree plot or Kaiser criterion (Eigenvalues > 1) are in line with the theory about the construct to be measured CFA: CFI or TLI or comparable measure > 0.95 *OR* RMSEA < 0.06 *OR* SRMR < 0.08 **IRT/Rasch**: No violation of *unidimensionality*: CFI or TLI or comparable measure > 0.95 *OR* RMSEA < 0.06 *OR* SRMR < 0.08 *AND* No violation of *local independence*: residual correlations among the items after controlling for dominant factor < 0.20 *OR* Q3s < 0.37 *AND* No violation of *monotonicity*: adequate looking graphs *OR* item scalability > 0.30 *AND* Adequate *model fit:* IRT: χ^2^ > 0.01 Rasch: infit and outfit mean squares ≥ 0.5 and ≤ 1.5 *OR* Z-standardized values >-2 and < 2
	?	Not enough information reported
	-	Criteria for ‘+’ not met
Internal consistency	+	At least low evidence for sufficient unidimensionality *AND* Cronbach’s alpha ≥ 0.70
	?	Criteria for “at least low evidence for sufficient unidimensionality” not met *OR* Evidence for insufficient unidimensionality *OR* Not enough information reported
	-	At least low quality evidence for sufficient unidimensionality *AND* Cronbach’s alpha < 0.70
Cross-cultural validity\ measurement invariance	+	No important differences found between group factors (such as age, gender, language) in multiple group factor analysis *OR* no important DIF for group factors (McFadden’s R^2^ < 0.02)
	?	Not enough information reported
	-	Important differences between group factors *OR* DIF was found
Reliability	+	ICC or (weighted) kappa or Pearson/Spearman correlation ≥ 0.70
	?	Not enough information reported
	-	ICC or (weighted) kappa or Pearson/Spearman correlation < 0.70
Measurement error	+	SDC or LoA < MIC
	?	MIC not defined *OR* not enough information reported
	-	SDC or LoA > MIC
Criterion validity	+	Correlation with gold standard ≥ 0.70 *OR* AUC ≥ 0.70
	?	Not enough information reported
	-	Correlation with gold standard < 0.70 *OR* AUC < 0.70
Hypotheses testing for construct validity	+	≥ 75% of the results is in accordance with predefined hypotheses
	?	No relevant results were found
	-	≥ 75% of the results deviates from predefined hypotheses
Responsiveness	+	≥ 75% of the results is in accordance with predefined hypotheses *OR* AUC ≥ 0.70
	?	No relevant results were found
	-	≥ 75% of the results deviates from predefined hypotheses *OR* AUC < 0.70

AUC = area under the receiver operating characteristic curve, CFA = confirmatory factor analysis, CFI = comparative fit index, CTT = classical test theory, DIF = differential item functioning, EFA = exploratory factor analysis, ICC = intraclass correlation coefficient, IRT = item response theory, LoA = limits of agreement, MIC = minimal important change, PCA = principal component analyses, RMSEA: Root Mean Square Error of Approximation, SEM = Standard Error of Measurement, SDC = smallest detectable change, SRMR: Standardized Root Mean Residuals, TLI = Tucker-Lewis index

### Step 6.4: summarize the results

If multiple studies on a measurement property of a PROM are available, it should be decided whether the results of all studies can be summarized. The main factor to consider is whether the results are consistent. If all results are either sufficient, insufficient, or indeterminate, the results can either be qualitatively summarized by describing the range of results, or the results can be statistically pooled across studies. In the manual we describe how to deal with inconsistent results.

### Step 6.5: rate the summarized results against criteria for good measurement properties

The summarized result of multiple studies on the same measurement property is rated using the same criteria for good measurement properties (Table 1).

### Step 6.6: grading the quality of the evidence (certainty assessment)

After all evidence is summarized and rated to determine whether a specific measurement property of the PROM under study is sufficient or not, the GRADE approach [25] can be used in the final sub-step of step 6 to grade the certainty of evidence. The evidence grading starts with assuming high quality evidence but will be downgraded to moderate, low, or very low quality evidence if there is: risk of bias (low quality of the studies), inconsistency (of the results of the studies), indirectness (evidence comes from different populations), or imprecision (wide confidence intervals or low sample sizes). As there are no registries for studies on the measurement properties of PROMs, the fifth factor used in the GRADE approach, publication bias (negative results are less often published), is not taken into account in the COSMIN guideline.

The grading of the three aspects of content validity (i.e. relevance, comprehensiveness and comprehensibility) is complex. Here, the PROM development, additional content validity studies (if available), and a rating from the review team need to be taken into account in the conclusion on the content validity of a PROM. Each of these factors provide information on the content validity, and it is not always clear whether to consider a study as a development study or a content validity study. The development consists of an elicitation phase in which the content of the PROM is determined (focusing on relevance and comprehensiveness), after which the PROM is developed. Only afterwards, the comprehensibility can be tested in a pilot study. In a content validity study each of the three aspect, that is the relevance, comprehensiveness, and comprehensibility, can be evaluated. How to deal with each of these studies when grading the evidence is precisely described in the manual. In Appendix 4 we explain and justify changes for grading the evidence for content validity compared to the previous version.

### Step 7: drawing conclusions

Based on the overview of the summarized results and quality of the evidence (certainty assessment) per measurement property per PROM, recommendations can be made in step 7. First, a PROM can be recommended to use (high quality evidence for sufficient results for all relevant measurement properties), recommended not to use (high quality evidence for insufficient results for at least one of the measurement properties), or no recommendation can yet be made (all other PROMs).

When the rational of the review was to recommend a PROM, based on the overview of the quality of the PROMs included, the most suitable instrument should be selected. If more than one PROM is assigned as ‘recommended for use’, the selection of the most suitable PROM can be based on the quality of the PROMs as well as on feasibility aspects (e.g. time aspects, budget constraints or availability in different languages) and interpretability aspects (e.g. availability of MIC values). If all PROMs are rated as ‘not recommended for use’, suggestions for improving the most promising PROM could be made. If none of the PROMs have enough information yet about their quality to be recommended for use, the PROM that has the (most) potential to be recommended for use can be selected. This PROM should have at least low quality evidence for sufficient content validity. In addition, a specific research agenda for this PROM can be proposed. In such an agenda it can be explained which measurement properties should be evaluated in the near future, and what kind of data is required to be able to draw conclusions on the specific measurement properties. In the manual we make recommendations for such a research agenda.

### Step 8: Comprehensive reporting

If authors of systematic reviews do not report sufficient details about how the review was conducted and how conclusions were drawn, users of these reviews are not able to judge the results, and safely use the review to select a PROM for their purpose [27]. Reporting guidelines aim to improve the completeness and transparency of research reports. To that end, the PRISMA-COSMIN for OMIs reporting guideline was published [22]. This reporting guideline can be used in step 8, as well as in step 1–3 to write the protocol of a systematic review. In addition to the PRISMA-COSMIN guideline, an Explanation & Elaboration document was published, containing many examples for good reporting [22].

### Supplementary materials

To further facilitate users of the COSMIN guideline, the original two user manuals (i.e. one for content validity and one for the other measurement properties) were combined and updated. In the new manual, each step of the systematic review is described in detail. An animated video was also developed in which the key steps of a systematic review of PROMS are explained. In addition, we provide an Excel working file, called the ‘COSMIN review management file’, which contains several worksheets for entering the extracted information on each PROM, the results of the included studies, the ratings of the quality of the studies (risk of bias), the ratings of the results of the measurement properties (against the criteria for good measurement properties), and the grading for the quality of the evidence (certainty assessment using the GRADE approach). In this file, also ready-made tables for reporting the review are provided. This file can be published as Supplementary material along the review article to provide readers the full details of the review. All materials are freely available on the COSMIN website (https://www.cosmin.nl).

## Discussion

There is an urgent need for more high quality systematic reviews of PROMS to facilitate better PROM selection and increased standardization of PROM use. High-quality systematic reviews have a well-defined research question, use a comprehensive search strategy, assess risk of bias of the included studies, evaluate the content validity of all included PROMs, and perform a transparent data synthesis and certainty assessment of the body of evidence per measurement property per PROM (subscale). We have updated the COSMIN guideline for systematic reviews of PROMs, including the COSMIN Risk of Bias checklist for PROMs and the COSMIN criteria for good measurement properties, and its accompanying manual. To improve the applicability of the guideline, we developed an animated video. To improve the transparency of a review, we developed the COSMIN review management file and we encourage users to publish this as a Supplement to the review. With these updates, we hope to better facilitate users who are conducting a systematic review on PROMs.

Conducting a systematic review of PROMs should not be taken lightly. Clearly defining the four key elements (that is, the scope of the review) will facilitate the conduct of the review, as well as the usefulness of the review for future users. We advise to seek assistance from clinical librarians to develop a comprehensive search strategy to identify all relevant studies to include in the review, and to translate the search strategy for use in different databases [28]. We also advise to include reviewers in the team with knowledge on PROM development and evaluation and at least some knowledge on the construct of interest and experience with the target population. Last, we advise to consider the feasibility of the review, to ensure high-quality of the work. Using the PRISMA-COSMIN for OMIs reporting guideline [22] ensures comprehensive reporting of the review.

COSMIN aims to improve the selection of measurement instruments by developing methodology, guidelines and other tools to facilitate researchers. The quality of future systematic reviews of PROMs can be improved by using the updated version of the COSMIN guideline for systematic reviews of PROMs. Better PROM selection and increased standardization of PROM use will lead to better research and clinical outcomes and ultimately improve health care.

## Electronic supplementary material

Below is the link to the electronic supplementary material.

Supplementary Material 1

Supplementary Material 2

Supplementary Material 3

Supplementary Material 4
