# Supplementary material for: COSMIN guideline for systematic reviews of patient-reported outcome measures version 2.0
Source: Qual Life Res. 2024 Aug 28;33(11):2929–39. doi: 10.1007/s11136-024-03761-6 (PMC11541334; doi:10.1007/s11136-024-03761-6)
Supplement: Supplementary file 1 — Supplementary Material 1 [file 11136_2024_3761_MOESM1_ESM.pdf]

COSMIN

COSMIN Tool VERSION 3.0

Risk of Bias checklist

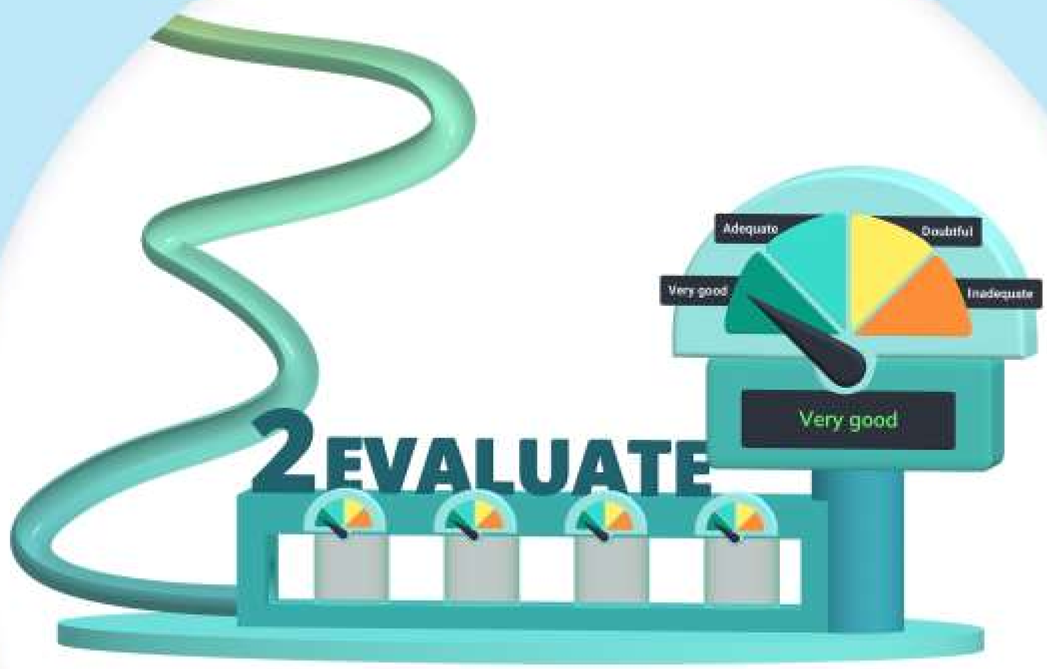

COSMIN Risk of Bias checklist

Date: June 2024

Contact
L.B. Mokkink, PhD
Amsterdam University Medical Centers
Department of Epidemiology and Data Science
Amsterdam Public Health research institute
P.O. box 7057
1007 MB Amsterdam
The Netherlands
Website: www.cosmin.nl
E-mail: w.mokkink@amsterdamumc.nl

How to site the COSMIN Risk of Bias Checklist

Please refer to the following study when using the COSMIN Risk of Bias Checklist version 3:

LB Mokkink, E Elsman, CB Terwee. (2024). The COSMIN guideline systematic reviews of Patient-Reported Outcome Measures (PROMs). Submitted for publication.

For details on how to use the COSMIN risk of Bias checklist see 'COSMIN guideline for conducting systematic reviews of Patient-Reported Outcome Measures (PROMs) – user manual' available from our website www.cosmin.nl.

Abbreviations used:

AUC – area under the receiver operating characteristic curve

CTT – classical test theory

DIF – differential item functioning

FA – factor analysis

ICC – intraclass correlation coefficient

IRT – item response theory

KR-20 - Kuder-Richardson formule 20

LoA – limits of agreement

MGCFA – multi-group confirmatory factor analysis

MI – measurement invariance

NA – not applicable

PCA – principal component analysis

PROM – patient-reported outcome measure

SDC – smallest detectable change

SE (ϑ) – standard error of the theta

SEM – standard error of measurement

1PL model – 1 parameter IRT model

2PL model – 2 parameter IRT model

Instructions

Tick the boxes that need to be completed for the article

	**COSMIN Risk of Bias checklist**
	Box 1. PROM development
	Box 2. Content validity
	Box 3. Structural validity
	Box 4. Internal consistency
	Box 5. Cross-cultural validity\Measurement invariance
	Box 6. Reliability
	Box 7. Measurement error
	Box 8. Criterion validity
	Box 9. Hypotheses testing for construct validity
	Box 10. Responsiveness

To assess the methodological quality of each study, i.e. assessing the risk of bias of the result of a study, you can use the corresponding COSMIN Risk of Bias box. You can complete each standard in the box, and use the 'worst score counts' method to determine the overall quality of a study (i.e. by taking the lowest rating of any standard in the box). For example, if for a reliability study one item in a box is rated as 'inadequate', the overall methodological quality of that reliability study is rated as 'inadequate'. The response option 'NA' (not applicable) is at issue for some standards. For example, when a study on structural validity is based on CTT, the standard on IRT is not applicable and this standard should not be considered in the "worst score counts"- rating for that specific study. For standards where this option is not at issue, these cells are grey and shouldn't be used.

Scope of the PROM	
1 Is a clear description provided of the construct to be measured?	○ Construct clearly described<br>○ Construct not clearly described
2 Is the origin of the construct clear: was a theory, conceptual framework or disease model used or clear rationale provided to define the construct to be measured?	○ Origin of the construct clear<br>○ Origin of the construct not clear
3 Is a clear description provided of the target population for which the PROM was developed?	○ Target population clearly described<br>○ Target population not clearly described
4 Is a clear description provided of the context of use	○ Context of use clearly described<br>○ Context of use not clearly described
5 What is the measurement model on which the PROM is based?	○ Reflective model<br>○ Formative model[1]<br>○ unclear

[1] If the scale is not based on a reflective model, unidimensionality or structural validity is not relevant.

Does the study concern unidimensionality or structural validity? [2]	○ unidimensionality<br>○ structural validity

[2] In a systematic review, it is helpful to make a distinction between studies where factor analysis is performed on each (sub)scale separately to evaluate whether the (sub)scales are unidimensional (unidimensionality studies) and studies where factor analysis is performed on all items of an instrument to evaluate the (expected) number of subscales in the instrument and the clustering of items within subscales (structural validity studies).

**Box 1. PROM development**						
1a. Concept elicitation study (relevance and comprehensiveness)		**very good**	**adequate**	**doubtful**	**inadequate**	**NA**
1	Was the concept elicitation study performed in a sample representing the target population for which the PROM was developed?	Study performed in a sample representing the target population	Assumable that the study was performed in a sample representing the target population, but not clearly described	Doubtful whether the study was performed in a sample representing the target population	Study not performed in a sample representing the target population	
2	Was an appropriate qualitative data collection method used to identify relevant items for a new PROM?	Widely recognized or well justified qualitative method used, suitable for the construct and study population	Assumable that the qualitative method was appropriate and suitable for the construct and study population, but not clearly described	Only quantitative (survey) method(s) used or doubtful whether the method was suitable for the construct and study population	Method used not appropriate or not suitable for the construct or study population	
3	Were skilled group moderators/interviewers used?	Skilled group moderators/ interviewers used	Group moderators /interviewers had limited experience or were trained specifically for the study	Not clear if group moderators /interviewers were trained or group moderators /interviewers not trained and no experience		NA

4	Were the group meetings or interviews based on an appropriate topic or interview guide?	Appropriate topic or interview guide	Assumable that the topic or interview guide was appropriate, but not clearly described	Not clear if a topic guide was used or doubtful if topic or interview guide was appropriate or no guide		NA
5	Were the group meetings or interviews recorded and transcribed verbatim?	All group meetings or interviews were recorded and transcribed verbatim	Assumable that all group meetings or interviews were recorded and transcribed verbatim, but not clearly described	Not clear if all group meetings of interviews were recorded and transcribed verbatim or recordings not transcribed verbatim or only notes were made during the group meetings/ interviews	No recording and no notes	NA
6	Was an appropriate approach used to analyse the data?	A widely recognized or well justified approach was used	Assumable that the approach was appropriate, but not clearly described	Not clear what approach was used or doubtful whether the approach was appropriate	Approach not appropriate	
7	Was at least part of the data coded independently?	At least 50% of the data was coded by at least two researchers independently	11-49% of the data was coded by at least two researchers independently	Doubtful if two researchers were involved in the coding or only 1-10% of the data was coded by at least two researchers independently	Only one researcher was involved in coding or no coding	Not applicab le
8	Was data collection continued until saturation was reached?	Evidence provided that saturation was reached	Assumable that saturation was reached	Doubtful whether saturation was reached	Evidence suggests that saturation was not reached	NA
9	For quantitative studies (surveys): was the sample size appropriate?	≥100	50-99	30-49	<30	NA

Other					
10 Were there any other important flaws in the design or methods of the study?	No other important methodological flaws		Other minor methodological flaws	Other important methodological flaws	

**1b. Pilot study (Cognitive interview study or other pilot test)** (comprehensibility)	**very good**	**adequate**	**doubtful**	**inadequate**	**NA**
Comprehensibility					
11 Was the pilot study performed in a sample representing the target population for which the PROM was developed?	Study performed in a sample representing the target population AND Sample was not included in the concept elicitation study	Assumable that the study was performed in a sample representing the target population but not clearly described, or sample was included in the concept elicitation study	Doubtful whether the study was performed in a sample representing the target population	Study not performed in a sample representing the target population	
12 Was the comprehensibility assessed of the PROM instructions, items, response options, and recall period?	Comprehensibility of the PROM instructions, items, response options, and recall period was assessed		Not clear if patients were asked about the comprehensibility of all items, response options, instructions, and recall period OR patients were not asked about the comprehensibility of the PROM instructions or the recall period	Patients were not asked about the comprehensibility of all items and response options	

13	Were all items tested in their final form?	All items were tested in their final form	Assumable that all items were tested in their final form, but not clearly described	Not clear if all items were tested in their final form	Items were not tested in their final form or items were not re-tested after substantial adjustments	
14	Was an appropriate qualitative method used?	Widely recognized or well justified qualitative method used	Assumable that the method was appropriate but not clearly described	Only quantitative (survey) method(s) used or doubtful whether the method was appropriate	Method used not appropriate	
15	Was each item tested in an appropriate number of patients?<br>For qualitative studies<br>For quantitative (survey) studies	 ≥7<br>≥50	 4-6<br>≥30	 <4 or not clear<br><30 or not clear		
16	Were skilled interviewers used?	Skilled interviewers used	Interviewers had limited experience or were trained specifically for the study	Not clear if interviewers were trained OR interviewers were not and had no experience		NA
17	Were the interviews based on an appropriate interview guide?	Appropriate topic or interview guide	Assumable that the topic or interview guide was appropriate, but not clearly described	Not clear if a topic guide was used or doubtful if topic or interview guide was appropriate OR no guide		NA
18	Were the interviews recorded and transcribed verbatim?	All interviews were recorded and transcribed verbatim	Assumable that all interviews were recorded and transcribed verbatim, but not clearly described	Not clear if all interviews were recorded and transcribed verbatim OR recordings not transcribed verbatim OR only notes were made during the interviews	No recording and no notes	NA

19	Was an appropriate approach used to analyse the data?	A widely recognized or well justified approach was used	Assumable that the approach was appropriate, but not clearly described	Not clear what approach was used OR doubtful whether the approach was appropriate	Approach not appropriate	
20	Were at least two researchers involved in the analysis?	At least two researchers involved in the analysis	Assumable that at least two researchers were involved in the analysis, but not clearly described	Not clear if two researchers were included in the analysis OR only one researcher involved in the analysis		
21	Were problems regarding the comprehensibility of the PROM instructions, items, response options, and recall period appropriately addressed by adapting the PROM?	No problems found OR problems appropriately addressed and PROM was adapted and re-tested if necessary	Assumable that there were no problems or that problems were appropriately addressed, but not clearly described	Not clear if there were problems OR doubtful if problems were appropriately addressed	Problems not appropriately addressed OR PROM was adapted but items were not re-tested after substantial adjustments	NA
Other						
22	Were there any other important flaws in the design or methods of the study?	No other important methodological flaws		Other minor methodological flaws	Other important methodological flaws	

Box 2. Content validity

2a. Asking patients about relevance

Design requirements		very good	adequate	doubtful	inadequate	NA
1	Was an appropriate method used to ask patients whether each item is <u>relevant</u> for their experience with the condition?	Widely recognized or well justified method used	Only quantitative (survey) method(s) used or assumable that the method was appropriate but not clearly described	Not clear if patients were asked whether <u>each</u> item is relevant OR doubtful whether the method was appropriate	Method used not appropriate OR patients not asked about the relevance of all items	
2	Was each item tested in an appropriate number of patients?					
	For qualitative studies	≥7	4-6	<4 or not clear		
	For quantitative (survey) studies	≥50	≥30	<30 or not clear		
3	Were skilled group moderators/interviewers used?	Skilled group moderators/ interviewers used	Group moderators /interviewers had limited experience or were trained specifically for the study	Not clear if group moderators /interviewers were trained OR group moderators /interviewers were not trained and had no experience		NA
4	Were the group meetings or interviews based on an appropriate topic or interview guide?	Appropriate topic or interview guide	Assumable that the topic or interview guide was appropriate, but not clearly described	Not clear if a topic guide was used OR doubtful if topic or interview guide was appropriate OR no guide		NA

5	Were the group meetings or interviews recorded and transcribed verbatim?	All group meetings or interviews were recorded and transcribed verbatim	Assumable that all group meetings or interviews were recorded and transcribed verbatim, but not clearly described	Not clear if all group meetings or interviews were recorded and transcribed verbatim OR recordings not transcribed verbatim OR only notes were made during the group meetings/ interviews	No recording and no notes	NA
Analyses						
6	Was an appropriate approach used to analyse the data?	A widely recognized or well justified approach was used	Assumable that the approach was appropriate, but not clearly described	Not clear what approach was used OR doubtful whether the approach was appropriate	Approach not appropriate	
7	Were at least two researchers involved in the analysis?	At least two researchers involved in the analysis	Assumable that at least two researchers were involved in the analysis, but not clearly described	Not clear if two researchers were included in the analysis OR only one researcher involved in the analysis		
Other						
8	Were there any other important flaws in the design or methods of the study?	No other important methodological flaws		Other minor methodological flaws	Other important methodological flaws	

2b Asking patients about comprehensiveness						
Design requirements		**very good**	**adequate**	**doubtful**	**inadequate**	**NA**
9	Was an appropriate method used for assessing the comprehensiveness of the PROM?	Widely recognized or well justified method used	Only quantitative (survey) method(s) used OR assumable that the method was appropriate but not clearly described	Doubtful whether the method was appropriate	Method used not appropriate	
10	Was the PROM tested in an appropriate number of patients?					
	For qualitative studies	≥7	4-6	<4 or not clear		
	For quantitative (survey) studies	≥50	≥30	<30 or not clear		
11	Were skilled group moderators/interviewers used?	Skilled group moderators/ interviewers used	Group moderators /interviewers had limited experience or were trained specifically for the study	Not clear if group moderators /interviewers were trained OR group moderators /interviewers were not trained and had no experience		NA
12	Were the group meetings or interviews based on an appropriate topic or interview guide?	Appropriate topic or interview guide	Assumable that the topic or interview guide was appropriate, but not clearly described	Not clear if a topic guide was used OR doubtful if topic or interview guide was appropriate OR no guide		NA

13 Were the group meetings or interviews recorded and transcribed verbatim?	All group meetings or interviews were recorded and transcribed verbatim	Assumable that all group meetings or interviews were recorded and transcribed verbatim, but not clearly described	Not clear if all group meetings or interviews were recorded and transcribed verbatim OR recordings not transcribed verbatim OR only notes were made during the group meetings/ interviews	No recording and no notes	NA
Analyses					
14 Was an appropriate approach used to analyse the data?	A widely recognized or well justified approach was used	Assumable that the approach was appropriate, but not clearly described	Not clear what approach was used OR doubtful whether the approach was appropriate	Approach not appropriate	
15 Were at least two researchers involved in the analysis?	At least two researchers involved in the analysis	Assumable that at least two researchers were involved in the analysis, but not clearly described	Not clear if two researchers were included in the analysis OR only one researcher involved in the analysis		
Other					
16 Were there any other important flaws in the design or methods of the study?	No other important methodological flaws		Other minor methodological flaws	Other important methodological flaws	

2c Asking patients about comprehensibility

Design requirements		very good	adequate	doubtful	inadequate	NA
17	Was an appropriate qualitative method used for assessing the <u>comprehensibility</u> of the PROM instructions, items, response options, and recall period?	Widely recognized or well justified qualitative method used	Assumable that the method was appropriate but not clearly described	Only quantitative (survey) method(s) used OR doubtful whether the method was appropriate OR not clear if patients were asked about the comprehensibility of the items, response options and recall period OR patients not asked about the comprehensibility of the PROM instructions or recall period	Method used not appropriate OR patients not asked about the comprehensibility of the items, response options, and recall period	
18	Was each item tested in an appropriate number of patients?					
	For qualitative studies	≥7	4-6	<4 or not clear		
	For quantitative (survey) studies	≥50	≥30	<30 or not clear		
19	Were skilled group moderators/interviewers used?	Skilled group moderators/ interviewers used	Group moderators /interviewers had limited experience or were trained specifically for the study	Not clear if group moderators /interviewers were trained OR group moderators /interviewers were not trained and had no experience		
20	Were the group meetings or interviews based on an appropriate topic or interview guide?	Appropriate topic or interview guide	Assumable that the topic or interview guide was appropriate, but not clearly described	Not clear if a topic guide was used or doubtful if topic OR interview guide was appropriate OR no guide		NA

21	Were the group meetings or interviews recorded and transcribed verbatim?	All group meetings or interviews were recorded and transcribed verbatim	Assumable that all group meetings or interviews were recorded and transcribed verbatim, but not clearly described	Not clear if all group meetings or interviews were recorded and transcribed verbatim OR recordings not transcribed verbatim OR only notes were made during the group meetings/ interviews	No recording and no notes	NA
Analyses						
22	Was an appropriate approach used to analyse the data?	A widely recognized or well justified approach was used	Assumable that the approach was appropriate, but not clearly described	Not clear what approach was used OR doubtful whether the approach was appropriate	Approach not appropriate	
23	Were at least two researchers involved in the analysis?	At least two researchers involved in the analysis	Assumable that at least two researchers were involved in the analysis, but not clearly described	Not clear if two researchers were included in the analysis OR only one researcher involved in the analysis		
Other						
24	Were there any other important flaws in the design or methods of the study?	No other important methodological flaws		Other minor methodological flaws	Other important methodological flaws	

2d. Asking professionals about relevance

Design requirements		very good	adequate	doubtful	inadequate	NA
25	Was an appropriate method used to ask professionals whether each item is relevant for the construct of interest?	Widely recognized or well justified method used	Only quantitative (survey) method(s) used OR assumable that the method was appropriate but not clearly described	Not clear if professionals were asked whether each item is relevant OR doubtful whether the method was appropriate	Method used not appropriate OR professionals not asked about the relevance of all items	
26	Were professionals from all relevant disciplines included?	Professionals from all required disciplines were included	Assumable that professionals from all required disciplines were included, but not clearly described	Doubtful whether professionals from all required disciplines were included OR relevant professionals were not included		
27	Was each item tested in an appropriate number of professionals?<br>For qualitative studies<br>For quantitative (survey) studies	 ≥7<br>≥50	 4-6<br>≥30	 <4 or not clear<br><30 or not clear		
Analyses						
28	Was an appropriate approach used to analyse the data?	A widely recognized or well justified approach was used	Assumable that the approach was appropriate, but not clearly described	Not clear what approach was used OR doubtful whether the approach was appropriate	Approach not appropriate	

29 Were at least two researchers involved in the analysis?	At least two researchers involved in the analysis	Assumable that at least two researchers were involved in the analysis, but not clearly described	Not clear if two researchers were included in the analysis OR only one researcher involved in the analysis		
Other					
30 Were there any other important flaws in the design or methods of the study?	No other important methodological flaws		Other minor methodological flaws	Other important methodological flaws	

**2e. Asking professionals about comprehensiveness**					
Design requirement	**very good**	**adequate**	**doubtful**	**inadequate**	**NA**
31 Was an appropriate method used for assessing the comprehensiveness of the PROM?	Widely recognized or well justified method used	Only quantitative (survey) method(s) used OR assumable that the method was appropriate but not clearly described	Doubtful whether the method was appropriate	Method used not appropriate	
32 Were professionals from all relevant disciplines included?	Professionals from all required disciplines were included	Assumable that professionals from all required disciplines were included, but not clearly described	Doubtful whether professionals from all required disciplines were included OR relevant professionals were not included		

33	Was the PROM tested in an appropriate number of professionals?<br>For qualitative studies<br>For quantitative (survey) studies	 ≥7<br>≥50	 4-6<br>≥30	 <4 or not clear<br><30 or not clear	
Analyses					
34	Was an appropriate approach used to analyse the data?	A widely recognized or well justified approach was used	Assumable that the approach was appropriate, but not clearly described	Not clear what approach was used OR doubtful whether the approach was appropriate	Approach not appropriate
35	Were at least two researchers involved in the analysis?	At least two researchers involved in the analysis	Assumable that at least two researchers were involved in the analysis, but not clearly described	Not clear if two researchers were included in the analysis OR only one researcher involved in the analysis	
Other					
36	Were there any other important flaws in the design or methods of the study?	No other important methodological flaws		Other minor methodological flaws	Other important methodological flaws

**2f. Asking professionals about comprehensibility**						
Design requirement		**very good**	**adequate**	**doubtful**	**inadequate**	**NA**
37	Was an appropriate method used for assessing the <u>comprehensibility</u> of the PROM instructions, items, response options, and recall period?	Widely recognized or well justified method used	Assumable that the method was appropriate but not clearly described	Only quantitative (survey) method(s) used OR doubtful whether the method was appropriate OR not clear if professionals were asked about the comprehensibility of all items, response options instructions, and recall period OR professionals were not asked about the comprehensibility of the PROM instructions or the recall period	Method used not appropriate OR professionals not asked about the comprehensibility of all items and response options	
38	Were professionals from all relevant disciplines included?	Professionals from all required disciplines were included	Assumable that professionals from all required disciplines were included, but not clearly described	Doubtful whether professionals from all required disciplines were included OR relevant professionals were not included		
39	Was each item tested in an appropriate number of professionals?<br>For qualitative studies<br>For quantitative (survey) studies	 ≥7<br>≥50	 4-6<br>≥30	 <4 or not clear<br><30 or not clear		

Analyses					
40	Was an appropriate approach used to analyse the data?	A widely recognized or well justified approach was used	Assumable that the approach was appropriate, but not clearly described	Not clear what approach was used OR doubtful whether the approach was appropriate	Approach not appropriate
41	Were at least two researchers involved in the analysis?	At least two researchers involved in the analysis	Assumable that at least two researchers were involved in the analysis, but not clearly described	Not clear if two researchers were included in the analysis OR only one researcher involved in the analysis	
Other					
42	Were there any other important flaws in the design or methods of the study?	No other important methodological flaws		Other minor methodological flaws	Other important methodological flaws

Box 3. Structural validity

Statistical methods	very good	adequate	doubtful	inadequate	NA
1 For CTT: Was exploratory or confirmatory factor analysis performed?	Confirmatory factor analysis performed	Exploratory factor analysis performed	Only PCA was performed	No exploratory or confirmatory factor analysis performed	NA
2 For IRT/Rasch: does the chosen model fit to the research question?	Chosen model fits well to the research question	Assumable that the chosen model fits well to the research question	Doubtful if the chosen model fits well to the research question	Chosen model does not fit to the research question	NA
3 Was the sample size included in the analysis adequate?	FA: 7 times the number of items in the tested model and ≥100	FA: at least 5 times the number of items in the tested model and ≥100; OR at least 6 times number of items in the tested model but <100	FA: 5 times the number of items in the tested model but <100	FA: < 5 times the number of items in the tested model	
	Rasch/1PL models: ≥ 200 subjects	Rasch/1PL models: 100-199 subjects	Rasch/1PL models: 50-99 subjects	Rasch/1PL models: < 50 subjects	
	2PL parametric IRT models OR Mokken scale analysis: ≥ 1000 subjects	2PL parametric IRT models OR Mokken scale analysis: 500-999 subjects	2PL parametric IRT models OR Mokken scale analysis: 250-499 subjects	2PL parametric IRT models OR Mokken scale analysis: < 250 subjects	
Other					
4 Were there any other important flaws in the design or statistical methods of the study?	No other important methodological flaws		Other minor methodological flaws (e.g. rotation method not described)	Other important methodological flaws (e.g. inappropriate rotation method)	

**Box 4. Internal consistency**					
Statistical methods	**very good**	**adequate**	**doubtful**	**inadequate**	**NA**
1 For continuous scores: Was Cronbach's alpha or omega calculated?	Cronbach's alpha, or Omega calculated		Only item-total correlations calculated	No Cronbach's alpha and no item-total correlations calculated	NA
2 For dichotomous scores: Was Cronbach's alpha or KR-20 calculated?	Cronbach's alpha or KR-20 calculated		Only item-total correlations calculated	No Cronbach's alpha or KR-20 and no item-total correlations calculated	NA
3 For IRT-based scores: Was standard error of the theta (SE (θ)) or reliability coefficient of estimated latent trait value (index of (subject or item) separation) calculated?	SE(θ) or reliability coefficient calculated			SE(θ) or reliability coefficient NOT calculated	NA
Other					
4 Were there any other important flaws in the design or statistical methods of the study?	No other important methodological flaws		Other minor methodological flaws	Other important methodological flaws	

**Box 5. Cross-cultural validity\Measurement invariance**					
Design requirements	**very good**	**adequate**	**doubtful**	**inadequate**	**NA**
1 Were the samples similar for relevant characteristics except for the group variable?	Evidence provided that samples were similar for relevant characteristics except group variable	Stated (but no evidence provided) that samples were similar for relevant characteristics except group variable	Unclear whether samples were similar for relevant characteristics except group variable	Samples were NOT similar for relevant characteristics except group variable	
Statistical methods					
2 Was an appropriate approach used to analyse the data?	A widely recognized or well justified approach was used	Assumable that the approach was appropriate, but not clearly described	Not clear what approach was used OR doubtful whether the approach was appropriate	Approach not appropriate	NA
3 Was the sample size included in the analysis adequate?	MGCFA: 7 times the number of items in the model and ≥100	5 times the number of items in the model and ≥100; OR 5-7 times the number of items in the model but <100	5 times the number of items in the model but <100	<5 times the number of items in the model	
	Regression analyses or IRT/Rasch based analyses: 200 subjects per group	150 subjects per group	100 subjects per group	< 100 subjects per group	
Other					
4 Were there any other important flaws in the design or statistical methods of the study?	No other important methodological flaws		Other minor methodological flaws	Other important methodological flaws	

**Box 6. Reliability**					
Design requirements	**very good**	**adequate**	**doubtful**	**inadequate**	**NA**
1 Were patients stable on the construct to be measured in the time between the repeated measurements?	Evidence provided that patients were stable	Assumable that patients were stable	Unclear if patients were stable	Patients were NOT stable	
2 Was the time interval between the repeated measurements appropriate?	Time interval appropriate		Doubtful if time interval was appropriate OR time interval was not stated	Time interval NOT appropriate	
3 Were the measurement conditions similar for the repeated measurements – except for the condition being evaluated?	Measurement conditions were similar (evidence provided)	Assumable that measurement conditions were similar	Unclear if measurement conditions were similar	Measurement conditions were NOT similar	
Statistical methods					
4 For continuous scores: Was the appropriate intraclass correlation coefficient (ICC) calculated?	$ICC_{agreement}$ was or can be calculated	($ICC_{consistency}$, Pearson or Spearman correlation coefficient was calculated, OR the ICC model or formula was not described)<br><br>WITH evidence provided that no systematic change between measurements has occurred<br><br>OR $ICC_{one-way}$ was calculated	($ICC_{consistency}$, Pearson or Spearman correlation coefficient was calculated OR the ICC model or formula was not described)<br><br>WITHOUT evidence provided that no systematic change between measurements has occurred	($ICC_{consistency}$, Pearson or Spearman correlation coefficient was calculated OR the ICC model or formula was not described)<br><br>WITH evidence provided that a systematic change between measurements has occurred	NA

5 For dichotomous scores: was kappa calculated?	Kappa calculated				NA
6 For nominal scores: was an unweighted kappa calculated?	Unweighted kappa calculated				NA
7 For ordinal scores: was a weighted kappa calculated?	Weighted kappa calculated and the weighting scheme was described	Kappa calculated, but weighting scheme not described	Unweighted Kappa calculated or unclear if weighting was applied		NA
Other					
8 Were there any other important flaws in the design or statistical methods of the study?	No other important methodological flaws		Other minor methodological flaws	Other important methodological flaws	

Box 7. Measurement error

Design requirements	**very good**	**adequate**	**doubtful**	**Inadequate**	**NA**
1 Were patients stable on the construct to be measured in the time between the repeated measurements?	Evidence provided that patients were stable	Assumable that patients were stable	Unclear if patients were stable	Patients were NOT stable	
2 Was the time interval between the repeated measurements appropriate?	Time interval appropriate		Doubtful if time interval was appropriate OR time interval was not stated	Time interval NOT appropriate	
3 Were the measurement conditions similar for the measurements – except for the condition being evaluated as a source of variation?	Measurement conditions were similar (evidence provided)	Assumable that measurement conditions were similar	Unclear if measurement conditions were similar	Measurement conditions were NOT similar	

Statistical methods						
4	For continuous scores: was the Standard Error of Measurement (SEM), Smallest Detectable Change (SDC) or Limits of Agreement (LoA) calculated?	$SEM_{agreement}$, $SEM_{one\text{-}way}$, $SDC_{agreement}$, or $SDC_{one\text{-}way}$ was or could be calculated	($SEM_{consistency}$ or $SDC_{consistency}$ or LoA was calculated OR the SEM/SDC model or formula was not described)<br><br>WITH evidence provided that no systematic change between the measurement has occurred	($SEM_{consistency}$, $SDC_{consistency}$, or LoA was calculated)<br><br>WITHOUT evidence provided that no systematic change between measurements has occurred	SEM calculated based on Cronbach's alpha or SD from another population<br><br>OR<br><br>($SEM_{consistency}$ $SDC_{consistency}$ or LoA calculated)<br>WITH evidence provided that systematic change has occurred	NA
5	For dichotomous/nominal/ordinal scores: was the percentage (positive and negative) agreement calculated?	% positive and negative agreement calculated	% agreement calculated			NA
Other						
6	Were there any other important flaws in the design or statistical methods of the study?	No other important methodological flaws		Other minor methodological flaws	Other important methodological flaws	

Box 8. Criterion validity

		very good	adequate	doubtful	inadequate	NA
Statistical methods						
1	For continuous scores: were correlations, or the AUC calculated?	Correlations or AUC calculated				NA
2	For dichotomous scores: were sensitivity and specificity determined?	Sensitivity and specificity calculated				NA
Other						
3	Were there any other important flaws in the design or statistical methods of the study?	No other important methodological flaws		Other minor methodological flaws	Other important methodological flaws	

Box 9. Hypotheses testing for construct validity

9a. Comparison with other outcome measurement instruments (convergent validity)

Design requirements		**very good**	**adequate**	**doubtful**	**inadequate**	**NA**
1	Is it clear what the comparator instrument(s) measure(s)?	Constructs measured by the comparator instrument(s) is clear			Constructs measured by the comparator instrument(s) is not clear	
2	Were the measurement properties of the comparator instrument(s) sufficient?	Sufficient measurement properties of the comparator instrument(s) in a population similar to the study population	Sufficient measurement properties of the comparator instrument(s) but not sure if these apply to the study population	Some information on measurement properties of the comparator instrument(s) in any study population	No information on the measurement properties of the comparator instrument(s), OR evidence for insufficient measurement properties of the comparator instrument(s)	
Statistical methods						
3	Were statistical methods adequate for the comparisons made?	Statistical methods applied were appropriate	Assumable that statistical methods were appropriate	Statistical methods applied NOT optimal	Statistical methods applied NOT appropriate	
Other						
4	Were there any other important flaws in the design or statistical methods of the study?	No other important methodological flaws		Other minor methodological flaws	Other important methodological flaws	

9b. Comparison between subgroups (discriminative or known-groups validity)					
Design requirements	**very good**	**adequate**	**doubtful**	**inadequate**	**NA**
5 Was an adequate description provided of important characteristics of the subgroups?	Adequate description of the important characteristics of the subgroups	Adequate description of most of the important characteristics of the subgroups	Poor of no description of the important characteristics of the subgroups		
Statistical methods					
6 Were statistical methods appropriate for the subgroups being compared?	Statistical methods applied were appropriate	Assumable that statistical methods were appropriate	Statistical methods applied NOT optimal	Statistical methods applied NOT appropriate	
Other					
7 Were there any other important flaws in the design or statistical methods of the study?	No other important methodological flaws		Other minor methodological flaws	Other important methodological flaws	

Box 10. Responsiveness

10a. Criterion approach (i.e. comparison to a gold standard)

		very good	adequate	doubtful	inadequate	NA
Statistical methods						
1	For continuous scores: were correlations between change scores, or the AUC calculated?	Correlations or AUC calculated				NA
2	For dichotomous scales: were sensitivity and specificity (changed versus not changed) determined?	Sensitivity and specificity calculated				NA
Other						
3	Were there any other important flaws in the design or statistical methods of the study?	No other important methodological flaws		Other minor methodological flaws	Other important methodological flaws	

**10b. Construct approach (i.e. hypotheses testing; comparison with other outcome measurement instruments)**					
Design requirements	**very good**	**adequate**	**doubtful**	**inadequate**	**NA**
4 Is it clear what the comparator instrument(s) measure(s)?	Constructs measured by the comparator instrument(s) is clear			Constructs measured by the comparator instrument(s) is not clear	
5 Were the measurement properties of the comparator instrument(s) sufficient?	Sufficient measurement properties of the comparator instrument(s) in a population similar to the study population	Sufficient measurement properties of the comparator instrument(s) but not sure if these apply to the study population	Some information on measurement properties of the comparator instrument(s) in any study population	NO information on the measurement properties of the comparator instrument(s) OR evidence of poor quality of comparator instrument(s)	
Statistical methods					
6 Were statistical methods appropriate for the comparisons being made?	Statistical methods applied appropriate	Assumable that statistical methods were appropriate	Statistical methods applied NOT optimal	Statistical methods applied NOT appropriate	
Other					
7 Were there any other important flaws in the design or statistical methods of the study?	No other important methodological flaws		Other minor methodological flaws	Other important methodological flaws	

**10c. Construct approach: (i.e. hypotheses testing: comparison between subgroups)**					
Design requirements	**very good**	**adequate**	**doubtful**	**inadequate**	**NA**
8 Was an adequate description provided of important characteristics of the subgroups?	Adequate description of the important characteristics of the subgroups	Adequate description of most of the important characteristics of the subgroups	Poor or no description of the important characteristics of the subgroups		
Statistical methods					
9 Were statistical methods appropriate for the subgroups being compared?	Statistical methods applied appropriate	Assumable that statistical methods were appropriate	Statistical method applied NOT optimal	Statistical method applied NOT appropriate	
Other					
10 Were there any other important flaws in the design or statistical methods of the study?	No other important methodological flaws		Other minor methodological flaws	Other important methodological flaws	

10d. Construct approach: (i.e. hypotheses testing: before and after intervention)

	very good	adequate	doubtful	inadequate	NA
Design requirements					
11 Was an adequate description provided of the intervention given?	Adequate description of the intervention		Poor description of the intervention	NO description of the intervention	
Statistical methods					
12 Were statistical methods appropriate for the before-after comparison being made?	Statistical methods applied appropriate	Assumable that statistical methods were appropriate	Statistical methods applied NOT optimal	Statistical methods applied NOT appropriate	
Other					
13 Were there any other important flaws in the design or statistical methods of the study?	No other important methodological flaws		Other minor methodological flaws	Other important methodological flaws	
