# Supplementary material for: COSMIN guideline for systematic reviews of patient-reported outcome measures version 2.0
Source: Qual Life Res. 2024 Aug 28;33(11):2929–39. doi: 10.1007/s11136-024-03761-6 (PMC11541334; doi:10.1007/s11136-024-03761-6)
Supplement: Supplementary file 2 — Supplementary Material 2 [file 11136_2024_3761_MOESM2_ESM.pdf]

Appendix 2. Changes made in the COSMIN Risk of Bias checklist version 3.0

The standards for PROM development (Box 1) and content validity (Box 2) were substantially changed. The standards for structural validity (box 4), reliability (box 5) and measurement error (box 6) were slightly adapted.

We made several changes in box 1. Items from the former version that referred to the scope of the PROM (box 1 standards 1-4, i.e. description of the construct to be measured, the target population and the context of use) were moved to step 5. The former items 14 (i.e. was a cognitive interview study or other pilot test conducted) and 16 (i.e. were patients asked about the comprehensibility of the PROM?) were deleted because these were not a standard for the quality of a study, but rather a screening item to decide whether the subsequent items are relevant. The former items 26-35 (assessing comprehensiveness in a pilot study) were deleted, because we argued that comprehensiveness is already addressed in the concept elicitation phase of the PROM development and we consider it not essential to repeat this in a pilot study.

In box 2 on content validity we have added six standards for assessing the quality of studies that ask professionals about comprehensibility of the PROM (items 37-42). Although it is most important to ask patients about the comprehensibility of a PROM, often also professionals are asked about the comprehensibility of the PROM. This could be considered relevant evidence for inclusion in a review, especially in cases where a pilot study in patients was not conducted. This box therefore now consists of 6 sub parts (see Appendix 2 Table 1).

Table 1 Sub parts of box 2 Content validity

2a. Asking patients about the relevance of the PROM items (standards (1-8)
2b. Asking patients about the comprehensiveness of the PROM (standards 9-16)
2c. Asking patients about the comprehensibility of the PROM (standards 17-24)
2d. Asking professionals about the relevance of the PROM items (standards 25-30)
2e. Asking professionals about the comprehensiveness of the PROM (standards 31-36)
2f. Asking professionals about the comprehensibility of the PROM (standards 37-42)

The boxes 3 and 4 started with a question about the type of study (i.e. unidimensionality or structural validity) and about whether the PROM consists of effect indicators (i.e. if the scale was based on a reflective model). As these questions do not refer to the quality of the study, but rather to the relevance of the study, we deleted these items from these boxes. In box 3 on Structural validity we have added a rating of 'doubtful' to item 1 (i.e. was exploratory or confirmatory factor analysis performed?) if only a principal component analysis was conducted.

The wording of the standards on reliability (box 6) and measurement error (box 7) was modified to be more clear and in line with the COSMIN Risk of Bias tool to assess studies on reliability and measurement error(1). For example, we now ask at standard 3 'Were the measurement conditions similar for the repeated measurements – except for the condition being evaluated?'. Also, the rating for the standard on the preferred statistical methods has been changed. A rating 'very good' for PROM scales with continuous scores now require that the $ICC_{agreement}$ (called ICC (2.1) by Shrout & Fleiss (2) (box 6 standard 4) or of the $SEM_{agreement}$ or $SDC_{agreement}$ (3) (box 7 standard 4) was or can be calculated. The standards for scales with other types of scores in box 6 (i.e. dichotomous, nominal or ordinal) have been simplified (see Appendix 1).

1. Mokkink LB, Boers M, van der Vleuten CPM, Bouter LM, Alonso J, Patrick DL, et al. COSMIN Risk of Bias tool to assess the quality of studies on reliability or measurement error of outcome measurement instruments: a Delphi study. BMC Medical Research Methodology. 2020;20(293).
2. Shrout PE, Fleiss JL. Intraclass Correlations: Uses in assessing rater reliability. Psychological Bulletin. 1979;86:420-8.
3. Mokkink LB, Eekhout I, Boers M, van der Vleuten CPM, de Vet HCW. Studies on Reliability and Measurement Error of Measurements in Medicine - From Design to Statistics Explained for Medical Researchers. Patient Relat Outcome Meas. 2023;14:193-212.
