# Supplementary material for: COSMIN guideline for systematic reviews of patient-reported outcome measures version 2.0
Source: Qual Life Res. 2024 Aug 28;33(11):2929–39. doi: 10.1007/s11136-024-03761-6 (PMC11541334; doi:10.1007/s11136-024-03761-6)
Supplement: Supplementary file 3 — Supplementary Material 3 [file 11136_2024_3761_MOESM3_ESM.pdf]

Appendix 3. Justification of changes to the criteria for good measurement properties

We now recommend to give an ‘indeterminate (?)’ rating for a measurement property if not enough information has been reported, although it is clear that a study on the specific measurement property has been conducted. We also added criteria for explorative factor analysis. A result on a study on internal consistency can now be rated with ‘sufficient (+)’ if there is at least low quality evidence for sufficient unidimensionality (instead of sufficient structural validity). This modification mainly has consequences for scales that are part of a multi-dimensional PROM. If the structural validity of the PROM is not sufficient, subscales of this PROM may still be unidimensional.
