# Supplementary material for: COSMIN guideline for systematic reviews of patient-reported outcome measures version 2.0
Source: Qual Life Res. 2024 Aug 28;33(11):2929–39. doi: 10.1007/s11136-024-03761-6 (PMC11541334; doi:10.1007/s11136-024-03761-6)
Supplement: Supplementary file 4 — Supplementary Material 4 [file 11136_2024_3761_MOESM4_ESM.pdf]

Appendix 4 – GRADE approach to determine the quality of the evidence for content validity (certainty assessment)

With the GRADE approach, the quality of the evidence is determined. The first factor that is taken into account in determining the quality (or certainty) of the evidence is the risk of bias. In general, when one study is of very good quality we do not downgrade. When drawing conclusions on content validity results from both the development as well as from additional content validity studies and the reviewers' own ratings can be considered. For relevance and comprehensiveness the concept elicitation phase, and any content validity studies in which specifically the relevance and comprehensiveness was evaluated are taken into account. For comprehensibility the pilot test and any content validity studies in which specifically comprehensibility was evaluated are considered. For grading the evidence, the reviews' ratings are not considered, and these ratings only lead to very low evidence.

The procedure for downgrading for risk of bias for content validity is slightly different than for other measurement properties (and changed compared to the previous version of the COSMIN guideline), specifically for the aspects relevance and comprehensiveness. The GRADE principle that we don't downgrade for risk of bias if one study is of very good quality also applies here. However, in the concept elicitation phase the content of the PROM is determined. The actual PROM (that is how the instructions, items or questions, and response options are formulated) is later developed. Consequently, the content validity of the PROM can't be evaluated yet in the concept elicitation phase. Therefore, we consider the evidence from the concept elicitation phase to be less strong for concluding on relevance and comprehensiveness than the evidence from a content validity study that evaluates these aspects. This is reflected in how to downgrade for risk of bias for these aspects.

To grade the evidence for comprehensibility, both the pilot study as well as content validity studies on comprehensibility (if available) are considered. We consider a pilot study and a content validity study of equal weight.

In addition to downgrading for risk of bias, also the other factors (inconsistency, indirectness and imprecision) are subsequently considered. A lower sample size is considered for downgrading the evidence of content validity for imprecision compared to other measurement properties. That is, if less than ten patients are involved we recommend to downgrade by two levels, and between 10 and 20 patients, we recommend to downgrade by one level.
